# Short-term effects of brachycephalic obstructive airway syndrome surgery on fitness and exercise in brachycephalic dogs

**DOI:** 10.3389/fvets.2025.1481717

**Published:** 2025-02-18

**Authors:** Juliette Goossens, Andrea Meyer-Lindenberg, Yury Zablotski, Maike Schroers

**Affiliations:** Clinic of Small Animal Surgery and Reproduction, Ludwig-Maximilians-University Munich, Munich, Germany

**Keywords:** dog, stress, saliva, fitness, BOAS

## Abstract

**Introduction:**

Brachycephalic obstructive airway syndrome (BOAS) significantly impacts the physical fitness of affected dogs. This study aimed to assess changes in physical fitness, as measured by vital signs and salivary cortisol and vasopressin concentrations, before and after BOAS surgery using a standardized fitness test.

**Methods:**

A prospective clinical study was conducted on 35 brachycephalic dogs, including 13 Pugs, 21 French Bulldogs, and one English Bulldog. A preliminary examination included clinical anamnesis and a general examination. Physical fitness was evaluated using a submaximal treadmill test consisting of three 5-minute runs followed by a recovery phase. Vital signs were monitored throughout the test and saliva samples were taken before, immediately after and 15 min after the test. Eighteen animals underwent surgery due to moderate to severe BOAS symptoms, while 17 animals that were free of symptoms were assigned to the control group.

**Results:**

One month post-OP (post-operatively), dogs that underwent BOAS surgery exhibited significant improvement in physical fitness based on vital signs. However, they remained significantly less fit than the control group. No statistically significant changes were observed in salivary cortisol or vasopressin concentrations before and after surgery.

**Discussion:**

BOAS surgery reduces clinical symptoms and improves physical fitness, but affected dogs continue to exhibit substantial limitations. The consistency of cortisol and vasopressin levels across both groups reinforces the hypothesis of a stress-induced HPA-axis dysfunction, yet the limited number of evaluable samples and external influences suggest that salivary stress hormones alone may not reliably indicate surgical success. Future studies should incorporate additional biomarkers and clinical assessments to better understand the physiological impact of BOAS and its treatment.

## 1 Introduction

Brachycephaly refers to the shortening of the skull in dogs and cats and is linked to respiratory conditions like BOAS (brachycephalic obstructive airway syndrome) (1–4). Brachycephalic breeds have a higher cephalic index, indicating a shorter and wider skull. This anatomical change contributes to the development of BOAS, as the compressed facial structure leads to inadequate space for soft tissues, increasing the risk of airway obstruction (1, 5, 6). These structural alterations include narrowing of the nostrils, extension of nasal turbinates into the nasopharynx, displacement and hyperplasia of the soft palate, a pharynx that is too short and too narrow, or enlargement and thickening of the tongue (6). Such abnormalities can result in stenosis or even partial obstruction of the upper airway (7). As a result, the resistance of the airflow increases during inspiration, requiring more effort. Increased airflow turbulence and negative pressure can lead to secondary issues like laryngeal or bronchial collapse (8, 9). Common respiratory symptoms include loud respiratory sounds such as stridor and stertor, increased inspiratory effort and dyspnea, which can lead to cyanosis, syncope, poor thermoregulation, and even death. Since the intensity of the symptoms often increases with age, BOAS is considered a progressive disease (5, 10).

In addition to respiratory problems, brachycephalic breeds are prone to other health issues due to their anatomical abnormalities, even if they are considered healthy. These issues can include gastrointestinal symptoms (e.g., regurgitation or vomiting), as well as ophthalmological problems (e.g., dry eyes and corneal ulcers) (6, 11, 12). Additionally, brachycephalic dogs are more susceptible to dermatological issues (e.g., dermatitis) due to their excess in skin folds, especially in the face, neck, and genital area. Due to their anatomical structure, brachycephalic breeds are also more prone to other issues such as dental abnormalities (e.g., malocclusion) and reproductive problems (e.g., dystocia) (6).

Respiratory difficulties, which often accompany BOAS, can lead to hypoxic stress, which activates the HPA axis (hypothalamic–pituitary–adrenal axis) and the sympathetic nervous system (13, 14). This releases hormones such as cortisol and vasopressin, so these hormones could provide clues to the stress state of a dog with BOAS. Elevated cortisol and vasopressin levels in saliva can indicate how the dogs are reacting to the physical strain of BOAS and help evaluate whether surgical intervention reduces their stress and improves their recovery (15–17). For example, dogs whose blood was drawn in an unfamiliar environment were found to have a greater stress response, based on increased serum cortisol levels, than dogs who were accustomed to having their blood drawn and therefore less stressed (18). Arginine vasopressin is a neuropeptide hormone and one of the primary activators of the HPA axis, which means it can be used as a behavioral and stress parameter. It is first synthesized in the hypothalamus and then secreted by the pituitary gland (15, 19). Chronic stress can also lead to changes in the HPA axis, affecting the production of cortisol and vasopressin (15). Cortisol and vasopressin levels can be measured in both saliva and blood. However, invasive methods like blood sampling can induce additional stress, potentially affecting measurements (20). To assess stress responses non-invasively and monitor recovery after surgery, measuring cortisol and vasopressin in saliva is valuable. These measurements are valuable for tracking changes over time without causing additional stress (17, 21, 22). Understanding how BOAS surgery influences these stress markers will clarify its impact on recovery. If the surgery successfully reduces stress levels, it may indicate an improvement in overall fitness and quality of life.

Symptoms of BOAS can be improved or even resolved by several factors. A conservative treatment approach consists of weight loss, exercise restriction and medication, which often includes the use of anti-inflammatory drugs, glucocorticoids, gastric acid-binding drugs or long-term oxygen supplementation (5, 23–25). In addition, using a harness instead of a collar and avoiding overheating, for example by moving walks to early morning or late evening, can reduce symptoms (5). Another option is surgical therapy aimed at reducing airway resistance (26). Depending on the cause, this may include correction of the nostrils, nasal vestibule and soft palate, laser-assisted turbinectomy, resection of everted laryngeal saccules and arytenoid lateralization (23). However, dogs with severe BOAS also have an increased risk of anesthesia (27, 28).

Given the lack of short-term studies examining the impact of BOAS surgery on physical fitness, this study aims to evaluate the immediate effects of surgical intervention by assessing physical fitness before and after the surgery using a fitness test to determine vital signs, as well as cortisol and vasopressin concentrations. The aim was to examine the extent to which BOAS symptoms can be alleviated with the help of surgery.

This study hypothesizes that surgical intervention for BOAS will improve physical fitness, alleviate respiratory symptoms, and potentially reduce stress levels as measured by hormone concentrations and aims to assess its impact on overall quality of life in brachycephalic dogs.

## 2 Materials and methods

All examinations and treatments were approved by the Ethics Committee of the Center for Clinical Veterinary Medicine, Faculty of Veterinary Medicine, Ludwig-Maximilian University, Munich, Germany (application 167–02-05-2019). The study was conducted from April 2021 to March 2023 at the Veterinary Clinic for Small Animals, Ludwig-Maximilians-University, Munich, Germany.

### 2.1 Animals

Dogs were included in the study based on the following inclusion criteria: The dogs had to belong to the breeds Pug, French Bulldog and English Bulldog. Dogs of other brachycephalic breeds were excluded. In addition, dogs had to be at least 12 months old and were excluded if they were younger. Dogs presenting with BOAS-associated respiratory problems and undergoing BOAS surgery were included in the study group if they had a BOAS-grade of 2 or 3. Dogs presenting for routine examination were included in the control group if there were no typical BOAS symptoms and the BOAS-grade was 0 or 1. Dogs with previous BOAS surgery were not included in the control group. Dogs that had difficulty walking due to orthopedic symptoms were also excluded from the study.

Of the 35 animals that participated in the fitness test, 17 were assigned to the control group. The BOAS surgery group consisted of 18 dogs. A total of 15 dogs had to be excluded from the study, of which 14 dogs had a high BOAS-grade but did not undergo BOAS surgery. The remaining patient had to be excluded due to non-attendance at the post-OP (post-operative) follow-up appointment.

BOAS-grade was assessed using the Cambridge University Group Brachycephalic Obstruction Airway Syndrome (BOAS) functional scoring system which is a scale used to assess the severity of respiratory impairment in brachycephalic dogs. Dogs were assigned grade 0 if there were no respiratory symptoms and no medical intervention is necessary. These dogs were considered BOAS-free. Grade 1 was assigned to dogs if there were respiratory symptoms, but they were so mild that they did not affect endurance and hence did not require medical intervention. Grades 2 and 3 are associated with medium- to high-grade respiratory problems, respectively, and veterinary treatment is recommended. Grade 2 dogs show clear signs of respiratory distress, particularly during physical exercise, which may affect their quality of life. Grade 3 dogs show severe signs of respiratory distress even at rest which suggests a high level of respiratory obstruction and may include difficulty with physical activity, and signs of cyanosis (29).

### 2.2 Study design

#### 2.2.1 Medical history

A medical history was taken before the start of the fitness test and included questions about the intensity and frequency of any respiratory sounds at rest and during sleep, which could be categorized as absent or present to a low or high degree. In addition, questions were asked about the intensity of potential movement intolerance, which could also be assessed as absent or slightly to highly present. The owner was also asked to rate the frequency of excitement in stressful situations as absent, rarely, sometimes, usually and always. Finally, information was obtained on the occurrence of syncope or shock and the presence of other diseases and medically necessary operations in the past.

#### 2.2.2 Study design

A clinical examination was carried out to assess general health. Vital signs such as respiratory rate, heart rate, mucosal color, capillary filling time and body temperature were measured. The breathing pattern was assessed and classified as normal, labored or dyspneic. Inspiratory effort was also assessed. Low effort was defined as a regular breathing cycle with minimal additional use of the diaphragm, moderate effort was defined as visibly increased use of the diaphragm and accessory respiratory muscles, and high effort was defined as significant, intense use of the diaphragm and accessory respiratory muscles. Signs of syncope or cyanosis (present or absent), nostril size (open or mild, moderate or severe stenosis), presence of stertor or stridor (present or absent) and auscultatory cardio-pulmonary sounds (normal or abnormal) of the study participants were determined. Based on these results, they were then categorized into the functional BOAS scoring system from 0 to 3 (28, 29). Dogs with no BOAS (BOAS 0) or very mild BOAS (BOAS 1) were assigned to the control group. Patients with moderate (BOAS 2) or severe (BOAS 3) BOAS presenting for surgical treatment were assigned to the study group.

#### 2.2.3 Treadmill test

The standardized fitness test was performed on a motorized treadmill (Kistler Instrumente GmbH, Munich, Germany) (30). The treadmill test began with a 15-min familiarization phase in the test room. This took place in a quiet environment with a room temperature between 20 and 24°C and a humidity between 31.0 and 48.4%. This was followed by 10 min on the treadmill to determine the individual running speed (from 0.8 m/s to 1.34 m/s) and a 15-min break. This was followed by the actual treadmill test as described by Mach et al. (30). This was performed in three running blocks of 5 min each. There was a one-minute break between two running blocks. Each running block began with a measurement of heart rate and respiratory rate and ended with a new measurement of heart rate. Heart rate was recorded using a commercially available heart rate belt (model H10 from Polar Electro GmbH, Büttelborn, Germany) strapped around the chest. The heart rate was measured again in the middle of each running block. The heart rate should increase by at least 40% of the baseline value during the test and the pace was adjusted if necessary. The heart rate should not exceed 200 beats per minute. A measurement exceeding this value will result in immediate termination of the treadmill test.

In addition, the presence of potential breath sounds, inspiratory effort, the presence of dyspnea and the dog’s willingness to run were assessed. The willingness to run was scored on a scale of 1 to 4 (1 - good willingness to run, 2 - moderate willingness to run, 3 - poor willingness to run, 4 - no willingness to run). The willingness to run was measured by behaviors such as pulling at the collar, attempting to run away or standing still. If there was insufficient willingness to run, the test was stopped, and the patient was excluded from the study. A sustained heart rate of at least 200 bpm and dyspnea in the dogs also resulted in termination of the treadmill test.

The fitness test began at minute 0 and ended at minute 17, followed by a 15-min recovery period in the examination room.

The dogs’ inspiratory effort was categorized as normal or low, moderate or high, and was assessed initially only while standing on the treadmill and then at the beginning of each break after a five-minute running block.

The assessment of potential dyspnea was graded as low- to high-grade, with low-grade dyspnea associated with signs of discomfort, medium-grade dyspnea with irregular breathing and signs of discomfort, and high-grade dyspnea with irregular breathing with clear signs of discomfort and difficulty breathing. For example, discomfort in dogs may be characterized by shallow and rapid breathing or panting, even at rest. There may also be restless behavior and changes in posture (31, 32).

To facilitate a direct comparison between the different groups, the recorded results were divided into two parts: The first part involved a comparison of the treadmill test with the measurement of heart rate and respiratory rate from pre- to post-OP, and the second part compared the results between the test and control groups.

#### 2.2.4 Saliva sampling

Immediately before [time point 1 (T1)] and immediately after the fitness test [time point 2 (T2)], saliva samples were collected using “Salivabio childrens swabs” saliva bottles from salimetrics®, LLC (Carlsbad, United States). Another saliva sample was collected 15 min after the end of the test [time point 3 (T3)]. The saliva vials were left in the oral cavity for 1.5–2 min at the junction of the oral mucosa and the maxillary gingiva at the level of the maxillary fourth premolar, and then secured in a sample tube provided for this purpose (33). All saliva samples were labeled and centrifuged at 470 rpm for 10 min at room temperature. After removal from the tubes, the salivettes were frozen upright at −20°C for 30 min. To minimize the influence of circadian variations, the samples were continuously collected at the same time of day.

#### 2.2.5 Quantitation of salivary vasopressin and cortisol

The concentration of cortisol in saliva was measured in the in-house laboratory using a commercially available ELISA kit from salimetrics®, LLC (Carlsbad, United States). In this kit, cortisol from standards and samples competes with horseradish peroxidase-conjugated cortisol for antibody binding sites on a microtiter plate. According to the salimetrics® manual (2021), the detection limit of the assay is 0.007 μg/dL (p. 16). Salivary vasopressin concentration was measured using a commercially available ELISA kit from Enzo Life Science® (Lörrach, Germany). This kit uses a microtiter plate coated with goat anti-rabbit IgG antibody with arginine vasopressin in combination with horseradish peroxidase. The detection limit of the assay is 2.84 pg./mL. All procedures were performed in the in-house laboratory in accordance with the product manual.

To facilitate a direct comparison between the different groups, the recorded results were divided into two parts, as in the evaluation of vital signs during the treadmill test: The first part involved a quantified comparison of cortisol and vasopressin concentrations from pre- to post-OP, and the second part compared the results between the study and control groups.

#### 2.2.6 Surgical treatment of BOAS

A staphylectomy was performed using the cut-and-sew technique. According to individual anatomical differences, the soft palate was resected and sutured in sections to remove excess tissue, so that it lies approximately in line with the base of the tongue without protruding into the airways during breathing (34–36). After resection, temporary extubation was done to check the length (34, 35).

Next, an alaplasty with wedge resection of the nasal wing was performed. The incision was made parallel to the edge of the nasal wing, and the tissue was sutured with single stitches (34, 35).

If the laryngeal saccules were everted, a sacculectomy was performed (37). If redundant nasal folds were present, these were also removed. Tonsils were generally not removed, and due to technical limitations no turbinectomy was performed.

BOAS surgery was performed under general anesthesia by administering propofol (1–4 mg/kg) for induction and isoflurane (1.5–2.5%) vaporized in 100% oxygen for maintenance. Using a flow-by system, every dog was pre-oxygenated prior to anesthesia for 10–15 min at a rate of 5 L/min with 100% oxygen and later intubated with an endotracheal tube. All dogs received peri-operative analgesia through the administration of intravenous methadone at a dose of 0.2 mg/kg and peri-operative fluid therapy was provided by intravenous administration of lactated ringer’s solution at 5–10 mL/kg/h. Post-OP analgesia was provided by intravenous administration of 0.01–0.02 mg/kg buprenorphine and 0.1 mg/kg Meloxicam which was reduced to only using Meloxicam orally for pain management after discharge. A CT scan was recommended prior to the procedure. However, at the owners’ requests, it was only performed in four dogs.

#### 2.2.7 Follow-up examination

Patients who underwent surgical treatment for BOAS (study group) were invited to a follow-up visit 4 weeks after surgery. A general examination and a standardized fitness test including the determination of cortisol and vasopressin in the dogs’ saliva were carried out again before and after the test.

### 2.3 Statistical analysis

Data analysis was performed using R 3.6.3 (2020-02-29). Results with a *p*-value <0.05 were considered statistically significant. Normality of the data was assessed using the Shapiro–Wilk test. For normally distributed data, generalized linear mixed-effects models (for cortisol) and robust generalized linear mixed models (for vasopressin) were used to predict cortisol and vasopressin levels over time for the surgery and control groups. All models included individual animals as a random effect fitted on the intercept. *p*-values after comparing pairwise categories were adjusted using the Tukey method for multiple comparisons.

For non-normally distributed data, the Mann–Whitney test and the Kruskal-Wallis test were used to analyze the heart rate and respiratory rate over time for the surgery and control groups.

Numerous charts and figures were created using Microsoft Excel to visually present the results.

### 2.4 Results

The study participants belonged to the breeds Pug, French Bulldog and English Bulldog (21 Pugs, 28 French Bulldogs and one English Bulldog) and were of both sexes, both neutered and intact. The mean age of the dogs was 4.1 years and ranged from 0.5 to 13 years, the mean BCS (Body Condition Score) was 5.9 and ranged from 3/9 to 9/9 (Table 1). In all surgical patients, a staphylectomy and an alaplasty was performed. In 10 patients, a sacculectomy was performed and two patients underwent a tonsillectomy in addition to these procedures. Redundant nasal folds were removed in two patients and one patient received a lateralization of the left arytenoid cartilage.

**Table 1:** Distribution of the study participants into the different categories breed, age, sex, BCS and BOAS-grade.

	Surgery group pre-OP	Surgery group post-OP	Control group
Participants	Total: 18		Total: 17
Breed	Pug: 7 FB^1^: 10 EB^2^: 1		Pug: 6 FB: 11 EB: 0
Age (years)	Pug: 1–10 (M^3^ = 5.1, SD^4^ = 3.5) FB: 1–11 (Md = 2, IQR = 2) EB: 3		Pug: 1–13 (Md^5^ = 2.5, IQR^6^ = 1) FB: 1–9 (M = 4.9, SD = 2.8)
Sex	Pug: 3 male, 4 female FB: 8 male, 2 female EB: 1 male		Pug: 1 male, 5 female FB: 2 male, 9 female
Weight (kg)	Pug: 6.7–10.1 (M = 6.4, SD = 2.7) FB: 11.4–15.0 (Md = 11.5, IQR = 10) EB: 39		Pug: 6.0–8.1 (M = 5.7, SD = 2.8) FB: 8.0–14.5 (M = 7.5, SD = 4.0)
BCS^7^	Pug: 5/9–9/9 (M = 7.0, SD = 1.3) FB: 4/9–7/9 (M = 5.8, SD = 0.9) EB: 8/9		Pug: 5/9–7/9 (M = 5.7, SD = 0.8) FB: 4/9–7/9 (M = 5.5, SD = 0.9)
BOAS^8^-grade	Pug: 1x grade 2, 6x grade 3 FB: 5x grade 2, 5x grade 3 EB: grade 2	Pug: 2x grade 1, 4x grade 2, 1x grade 3 FB: 2x grade 1, 5x grade 2, 3x grade 3 EB: grade 2	Pug: all grade 1 FB: all grade 1

^1FB = French Bulldog, 2EB = English Bulldog, 3M = Mean, 4SD = Standard Deviation, 5Md = Median, 6IQR = Interquartile Range, 7BCS = Body Condition Score, 8BOAS = Brachycephalic obstructive airway syndrome.^

#### 2.4.1 Medical history

**Figure 1:**
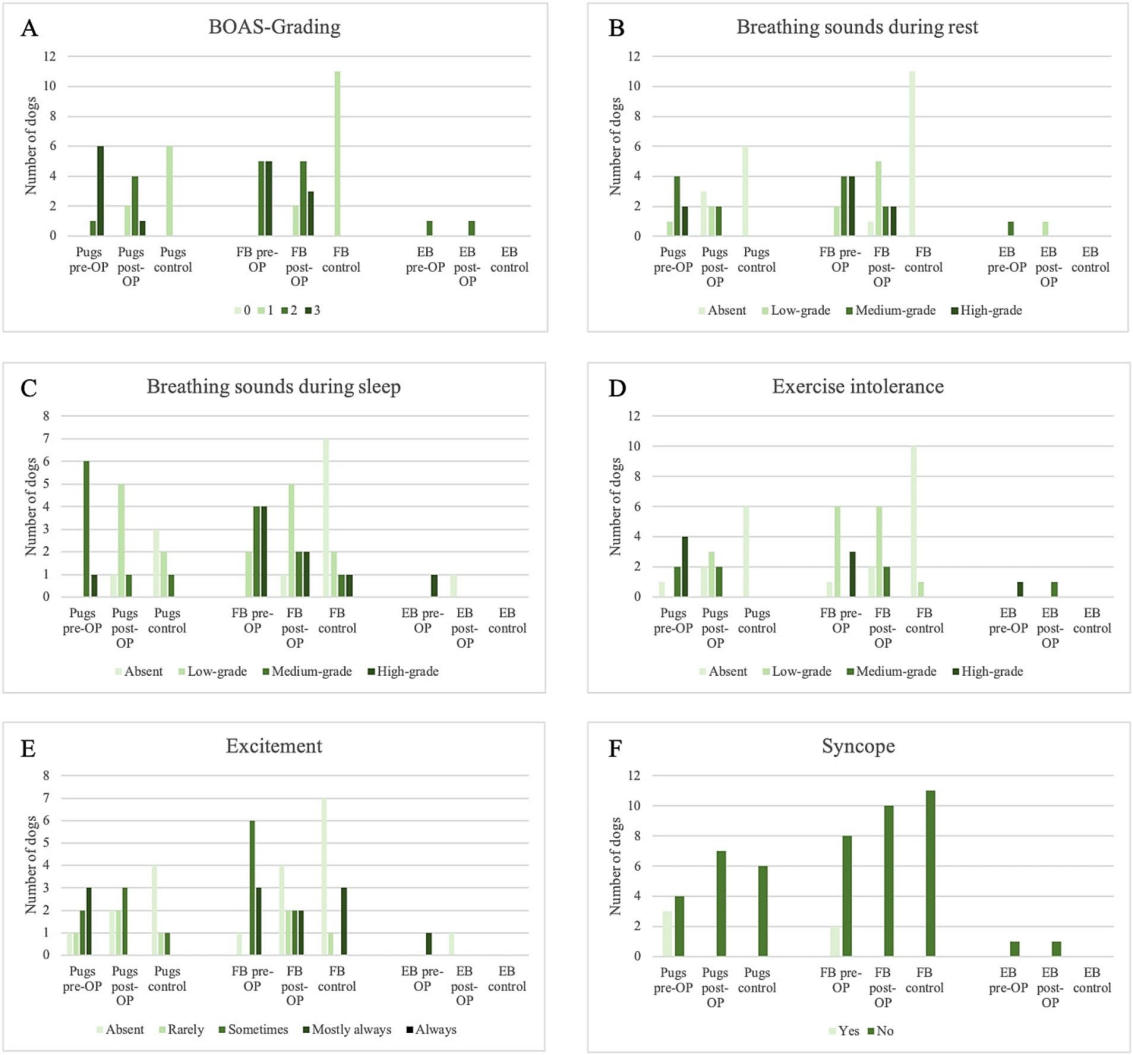
Overview of Medical History within the surgery group pre-OP and post-OP and the control group. **(A)** Change in BOAS-grading in the different dog breeds within the surgical group and the control group. **(B)** Change in respiratory sounds at rest in the different dog breeds within the surgery group and the control group. **(C)** Change in respiratory sounds during sleep in the different dog breeds within the surgery group and the control group. **(D)** Change in exercise intolerance in the different dog breeds within the surgery group and the control group. **(E)** Change in the excitement tendency in the different dog breeds within the surgery group and the control group. **(F)** Change in syncope incidence in the different dog breeds within the surgical group and the control group.

The surgical group consisted of 18 patients, of which seven dogs were Pugs (aged one to 10 years, with a BCS of 5/9 to 9/9), 10 dogs were French Bulldogs (aged one to 11 years, with a BCS of 4/9 to 7/9), and one dog was an English Bulldog (aged 3 years, BCS 8/9). Before surgery, seven dogs were classified as BOAS-grade 2 and 11 dogs were classified as BOAS-grade 3 (Table 1). Post-OP, these numbers changed to five dogs with BOAS-grade 1, nine dogs with BOAS-grade 2 and four dogs with BOAS-grade 3 (Figure 1A). Of the Pugs, three dogs improved by one BOAS-grade (from grade 3 to grade 2) and two improved by two BOAS-grades (from grade 3 to grade 1). Two Pugs did not change their grades (one Pug remained at grade 2 and one Pug remained at grade 3). Of the French Bulldogs, two dogs improved by one BOAS-grade (one dog from grade 3 to grade 2 and one dog from grade 2 to grade 1) and one French Bulldog improved by two BOAS-grades (grade 3 to grade 1). Seven French Bulldogs did not change grade (three dogs remained in grade 3 and four dogs remained in grade 2). The BOAS-grade of the English Bulldog remained unchanged post-OP compared to pre-OP (pre-operative) at grade 2. BCS remained the same post-OP as pre-OP in all patients.

Overall, 44.4% of the dogs improved by at least one BOAS-grade, of which the BOAS surgery even resulted in an improvement of two BOAS-grades in 16.7%. 55.6% remained the same in BOAS-grade, of which 22.2% remained at grade 3 and 33.3% remained at grade 2.

Pre-OP, all 18 owners reported that their dog made respiratory sounds at rest (Figure 1B). Of these, nine (four Pugs, one English Bulldog and four French Bulldogs) and six (two Pugs and four French Bulldogs) reported that the noises were moderate or severe. In 13 patients, there was an improvement in respiratory symptoms after BOAS surgery (Figure 1B): Two French Bulldogs improved from severe to moderate respiratory symptoms and six dogs improved from medium- to low-grade respiratory symptoms (three French Bulldogs, two Pugs and one English Bulldog). Two dogs had complete resolution of low (one Pug and one French Bulldog) and high (two Pugs) respiratory sounds, and one French Bulldog had a reduction from high to low. In four patients, there was no change in breath sounds: one French Bulldog did not improve and retained high-grade respiratory sounds, and two Pugs and one French Bulldog did not improve medium-grade respiratory sounds. The owner of one French Bulldog stated that the respiratory sound had worsened (from medium- to high-grade) after surgery. This animal underwent rhinoplasty, staphylectomy, tonsillectomy and resection of inverted sacculi. A CT scan revealed the presence of aberrant conchae in the ventral nasal passage. Four dogs (three Pugs and one French Bulldog) were completely free of wheezing after surgery according to the owners’ survey. The intensity of stridor was reduced to low-grade in six (three French Bulldogs, two Pugs and one English Bulldog) of the remaining patients with moderate stridor and in one French Bulldog with a high-grade stridor. In two Pugs, the high-pitched wheezing disappeared completely.

All owners reported that their dog made respiratory sounds during sleep prior to surgery (Figure 1C). Of these, 10 and six reported that the noise was of medium- and high-grade, respectively. In 13 patients, breathing symptoms during sleep also improved after BOAS surgery: Two French Bulldogs improved from high- to medium-grade, six dogs (four Pugs and two French Bulldogs) improved from medium- to low-grade and one English Bulldog improved from high-grade to absent. One Pug and one French Bulldog each had their respiratory problems reduced from high- to low-grade, and the medium-grade respiratory problems of one French Bulldog and one Pug were completely resolved. Three patients had no change in respiratory sounds (2x low-grade and 1x high-grade) and one owner stated that respiratory sounds during sleep had worsened from medium- to high-grade after surgery. This is the same patient whose respiratory sounds were also worse at rest according to the owner. Radiographic examination revealed tracheal hypoplasia with a tracheal index of 0.175.

In addition, all 18 patients were affected by exercise intolerance according to their owners (Figure 1D). Eleven owners rated this as medium- to high-grade. In 13 dogs this was improved by BOAS surgery, while four patients showed no change. An improvement in exercise intolerance was equated with increased endurance, which could be defined as longer walks at a faster pace. According to the owners, six dogs were completely free of exercise intolerance after surgery. One owner reported that exercise intolerance had worsened after surgery. This was evidenced by earlier exhaustion during walks and play. This patient underwent rhinoplasty, staphylectomy and resection of redundant nasal folds. In one patient with high-grade exercise intolerance, this was reduced to a low-grade.

Seventeen dogs were reported by their owners to become rapidly agitated in stressful situations, which manifested as labored breathing or panting (Figure 1E). Twelve patients exhibited this behavior frequently or constantly. Post-OP, seven of these dogs no longer exhibited these symptoms.

Seven of the 18 dogs had at least one episode of syncope during their lifetime (Figure 1F). There was no recurrence in any of the patients between surgery and the four-week follow-up.

The control group consisted of 17 dogs, six of which were Pugs (aged one to 13 years with a BCS of 5/9 to 7/9) and 11 of which were French Bulldogs (aged one to 9 years with a BCS of 4/9 to 7/9) (Table 1). All dogs were classified as BOAS-grade 1 and had never undergone BOAS surgery. At the time of the anamnesis interview, all owners reported that their dog had no respiratory sounds at rest. Of these, seven owners reported that their dog had respiratory sounds during sleep, five of which were classified as low-, one as medium and one as high-grade. Of the seven dogs, four were French Bulldogs and three were Pugs. Only one owner reported that their dog had a mild degree of exercise intolerance. The other dogs had no problems. Five of the 17 dogs showed mild excitement in stimulating situations, characterized by slightly faster and deeper breathing. Three of these dogs displayed this response more frequently.

In addition, nine of the 17 control dogs had undergone at least one surgery in the past. Five of these animals had been neutered, one of which had also undergone dental cleaning. Two animals underwent cesarean sections, one animal was operated on for a unilateral patella dislocation and one dog was operated on for a granuloma. None of these animals had any pain or musculoskeletal limitations at the time of the study. In total, the owners of three dogs stated that their animal had no complaints and had never been operated on.

#### 2.4.2 Treadmill examination with measurement of vital signs

##### 2.4.2.1 Comparison between brachycephalic dogs with BOAS pre- and post-OP

###### 2.4.2.1.1 Heart rate

**Figure 2:**
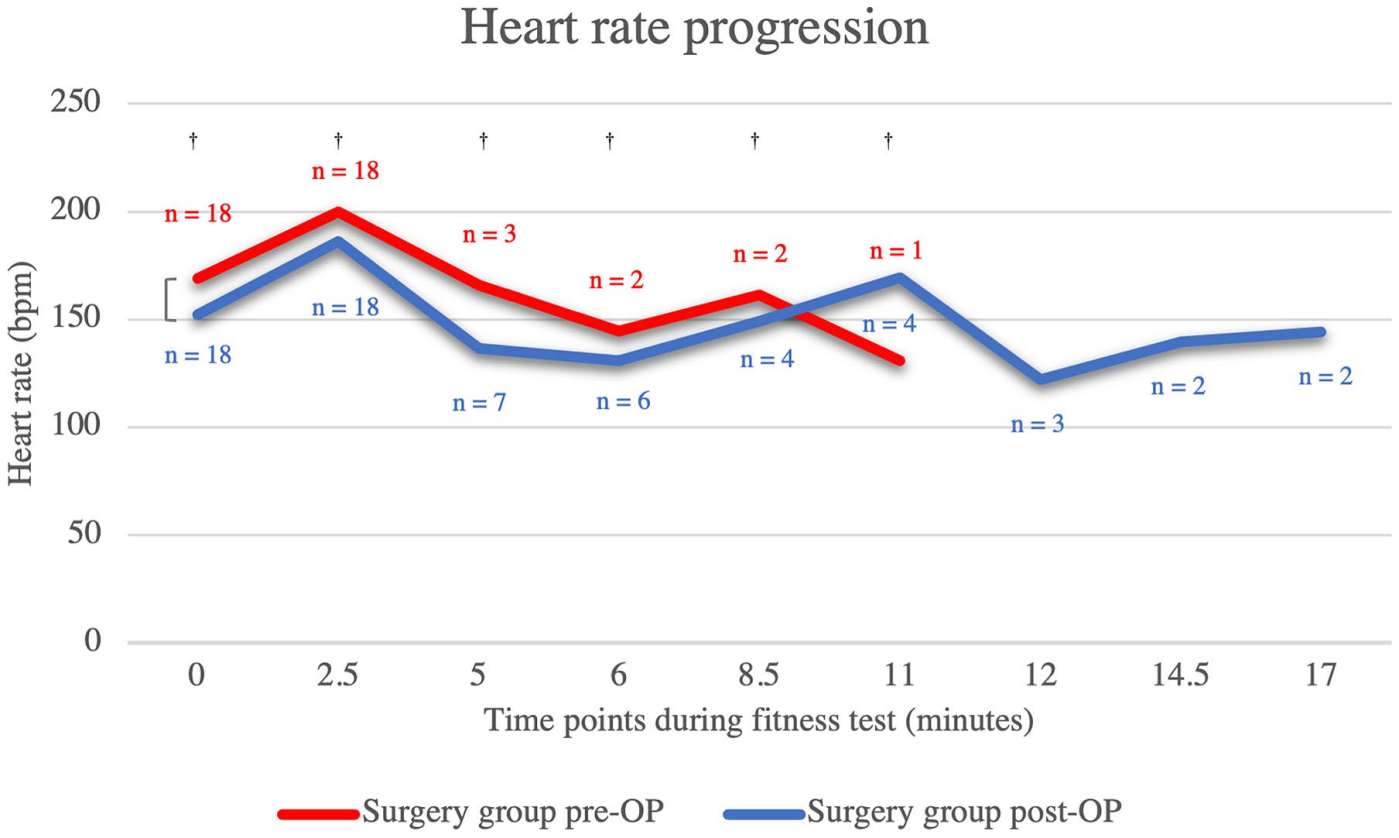
Comparison of the heart rate within the surgical group before and after the BOAS surgery during the fitness test. The pre-OP measurement shows that the patients almost always had a higher respiratory rate than post-OP, although the difference were never statistically significant (minute 0: *p* = 0.11, minute 2.5: *p* = 0.34, minute 5: *p* = 1.00, minute 6: *p* = 0.82, minute 8.5: *p* = 1.00, minute 11: *p* = 0.29). The graph showing the heart rates of the pre-OP animals ends at minute 11, as the fitness test could not be continued any longer in any of the pre-OP animals. Pre-OP: minute 0: median = 169, IQR = 33, minute 2.5: median = 200, IQR = 16, minute 5: mean = 166, SD = 34.7707, minute 6: mean 144.5, SD = 27.5772, minute 8.5: mean = 161.5, SD = 40.3051, minute 11: mean = 131, SD = 0. Post-OP: minute 0: mean = 152.28, SD = 26.0131, minute 2.5: mean = 185.94, SD = 27.4965, minute 5: mean = 136.71, SD = 17.7643, minute 6: mean = 131, SD = 8.641, minute 8.5: mean = 149, SD = 3.6515, minute 11: mean = 169.25, SD = 7.2744, minute 12: mean = 122, SD = 13, minute 14.5: mean = 139.5, SD = 7.7782, minute 17: mean = 144, SD = 11.3137. ^†^*p* ≥ 0.05.

Compared to the post-OP measurement of heart rate, the pre-OP measurement shows that the patients almost always had a higher respiratory rate, although this was not statistically significant (minute 0: *p* = 0.11, minute 2.5: *p* = 0.34, minute 5: *p* = 1.00, minute 6: *p* = 0.82, minute 8.5: p = 1.00, minute 11: *p* = 0.29; Figure 2; Supplementary Figure S1). Only at minute 11 was the mean heart rate of the pre-OP animals higher than that of the post-OP animals. However, at this time the pre-OP group consisted of only one animal, whereas the post-OP group consisted of four animals, as the fitness test had to be terminated prematurely in the surgical animals. The graph showing the heart rates of the pre-OP animals ends at minute 11, as the fitness test could not be continued any longer in any of the pre-OP animals. All animals in the surgical group remained within the accepted maximum heart rate of 200 bpm at 0 min and 2.5 min. At 5 min, the post-OP group consisted of only seven animals, six of which had previously terminated the test pre-OP. At the 6-min and 8.5-min time points, six and four post-OP animals were running on the treadmill, respectively, two of which had to abandon the fitness test prior to these time points pre-OP. At the 11-min and 12-min time points, four and three animals, respectively, were running post-OP, with three of these dogs having to abandon the fitness test prior to these time points pre-OP. Only two dogs were still running at 14 min and 17.5 min post-OP, both of whom were able to complete the entire test pre-OP.

###### 2.4.2.1.2 Respiratory rate

Both the pre- and post-OP measurements showed that the median respiratory rate was consistently 200 breaths per minute. In this study, panting was equated with a respiratory rate of 200 breaths per minute. As the pre-OP fitness test had to be terminated prematurely in all animals, the graph ends at minute 11, after which the respiratory rate of the post-OP animals remained the same. No statistically significant differences were found (minute 0: *p* = 0.71, minute 5: p = NA, minute 11: p = NA, minute 17: *p* = 1.00).

##### 2.4.2.2 Comparison between brachycephalic dogs with BOAS pre-OP and brachycephalic dogs without BOAS

###### 2.4.2.2.1 Heart rate

**Figure 3:**
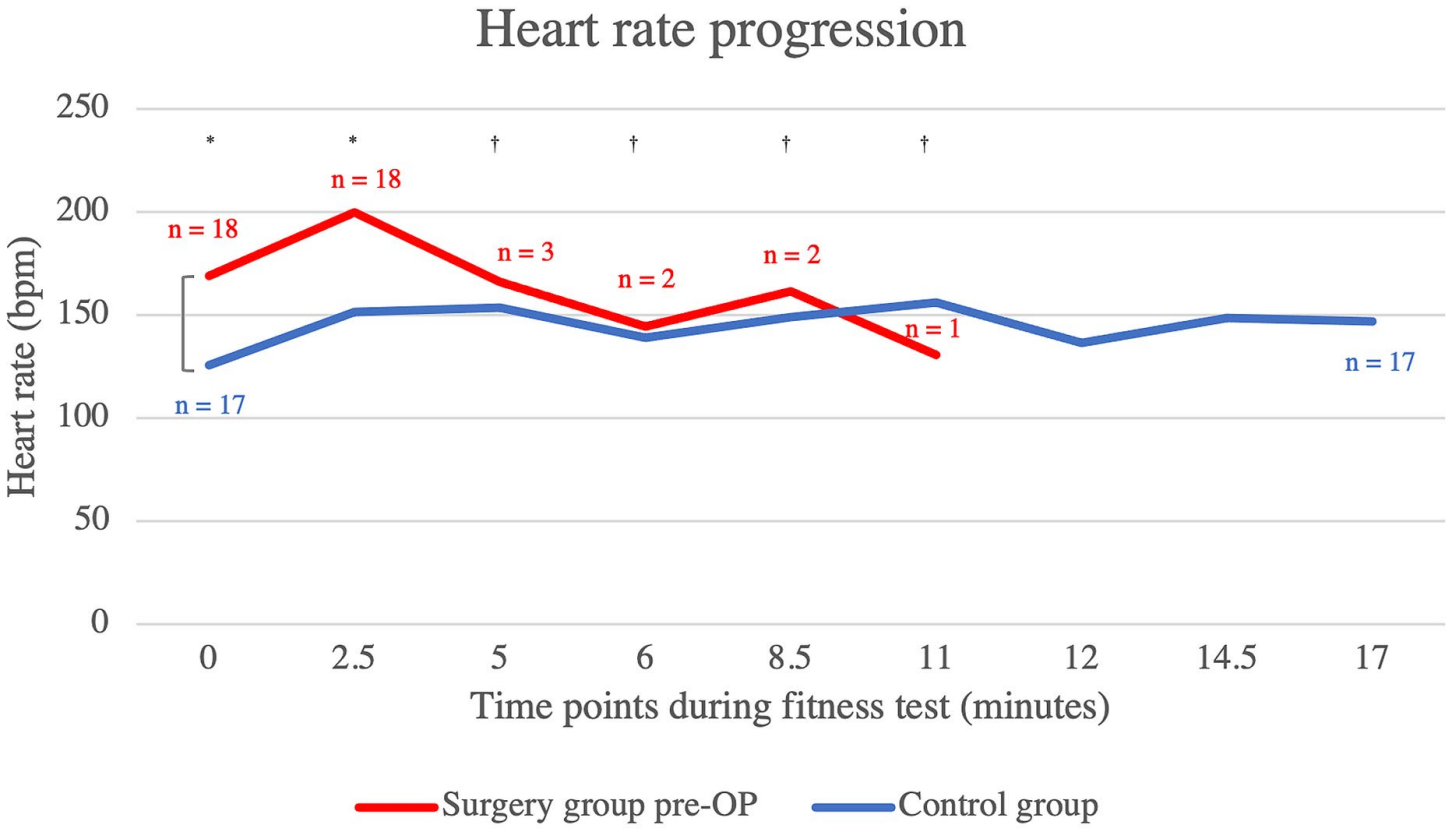
Comparison of the heart rate within the control group animals and the surgery group animals before BOAS surgery during the fitness test. The pre-OP measurement shows that the patients almost always had a higher respiratory rate than the control animals with the differences being statistically significant up to the fifth minute (minute 0: *p* = 0.00122, minute 2.5: p = 0.00122, minute 5: *p* = 0.48, minute 6: *p* = 0.58, minute 8.5: p = 1.00, minute 11: *p* = 0.14). The graph showing the heart rates of the pre-OP animals ends at minute 11, as the fitness test could not be continued any longer in any of the pre-OP animals. Pre-OP: minute 0: median = 169, IQR = 33, minute 2.5: median = 200, IQR = 16, minute 5: mean = 166, SD = 34.7707, minute 6: mean 144.5, SD = 27.5772, minute 8.5: mean = 161.5, SD = 40.3051, minute 11: mean = 131, SD = 0. Post-OP: minute 0: median = 126, IQR = 28.5, minute 2.5: mean = 151.76, SD = 21.1232, minute 5: mean = 153.53, SD = 16.5685, minute 6: mean = 139, SD = 21.8117, minute 8.5: mean = 149, SD = 13.1529, minute 11: mean = 156, SD = 13.4676, minute 12: mean = 136.47, SD = 17.8435, minute 14.5: mean = 148.65, SD = 15.0122, minute 17: median = 147, IQR = 22.5. ^*^p < 0.05, ^†^*p* ≥ 0.05.

The patients who underwent BOAS surgery showed an almost constantly higher heart rate in the pre-OP examination compared to the control animals, which was statistically significant up to the fifth minute (minute 0: *p* = 0.00122, minute 2.5: p = 0.00122, minute 5: *p* = 0.48, minute 6: *p* = 0.58, minute 8.5: *p* = 1.00, minute 11: *p* = 0.14) (Figure 3). Only at minute 11 is the median heart rate of the surgical group lower than that of the control animals. However, at this time the pre-OP group consisted of only one animal, whereas the control group consisted of 17 animals. The graph showing the heart rates of the pre-OP animals ends at minute 11, as it was not possible to continue the fitness test for any of these animals.

###### 2.4.2.2.2 Respiratory rate

**Figure 4:**
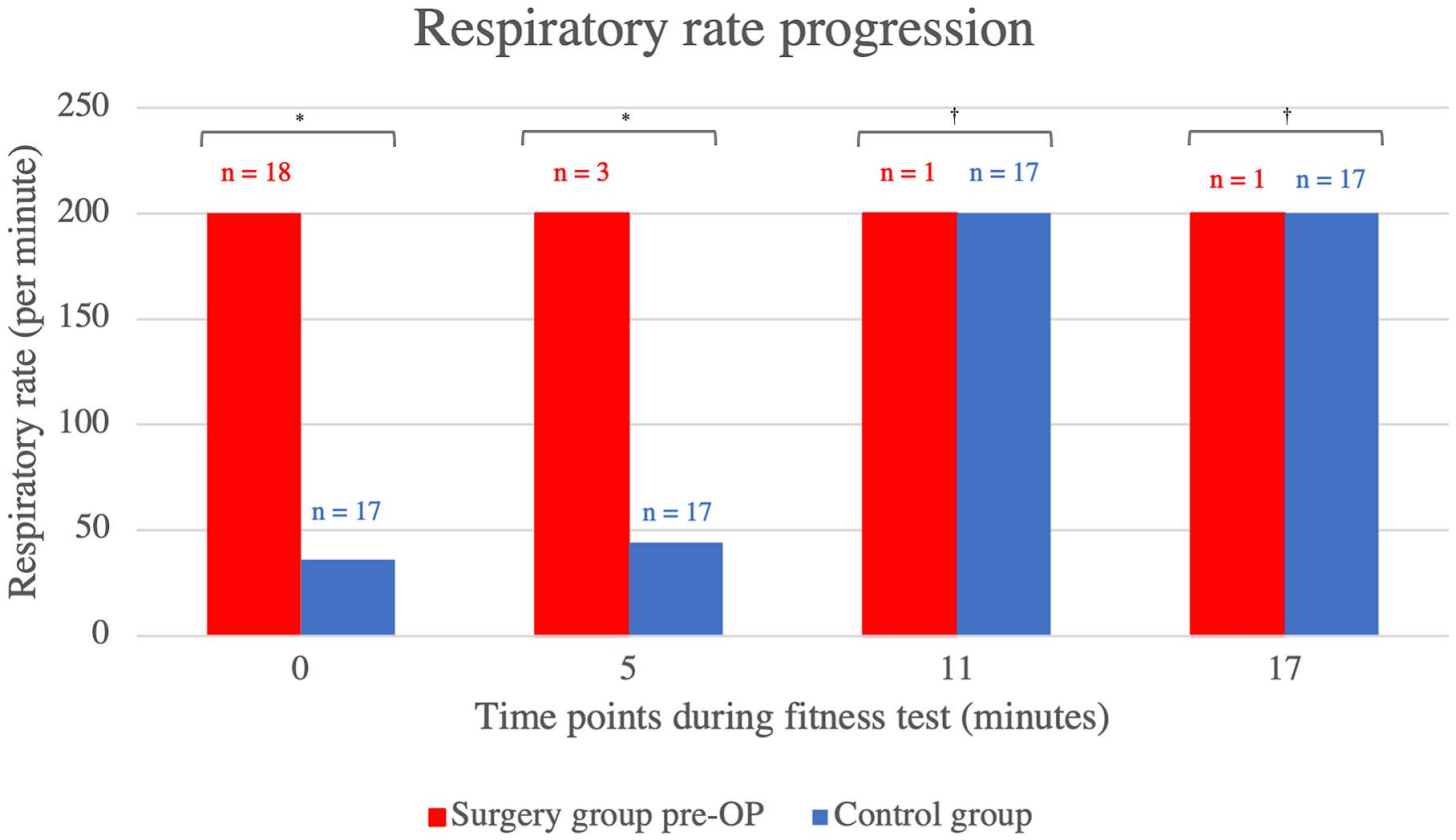
Comparison of the respiratory rate within the control group animals and surgery group animals before BOAS surgery during the fitness test. The pre-OP measurement shows that the patients had a consistently higher respiratory rate than the control animals with the differences being statistically significant up to the eleventh minute (minute 0: *p* = 0.01, minute 5: *p* = 0.04, minute 11: *p* = 0.64, minute 17: *p* = 0.73). The graph showing the heart rates of the pre-OP animals ends at minute 11, as the fitness test could not be continued any longer in any of the pre-OP animals. Pre-OP: minute 0: median = 200, IQR = 164, minute 5: median = 200, IQR = 0, minute 11: median = 200, IQR = 0, minute 17: median = 200, IQR = 0. Control: minute 0: median = 36, IQR = 12, minute 5: median = 44, IQR = 160, minute 11: median = 200, IQR = 152, minute 17: median = 200, IQR = 76. ^*^p < 0.05, ^†^*p* ≥ 0.05.

In terms of respiratory rate, the patient group that underwent BOAS surgery showed a consistently higher respiratory rate in the pre-OP fitness test compared to the control animals, which was statistically significant up to the eleventh minute (minute 0: *p* = 0.01, minute 5: *p* = 0.04, minute 11: *p* = 0.64, minute 17: *p* = 0.73; Figure 4). As the fitness test had to be terminated prematurely in all animals pre-OP, the graph ends at minute 11, at which point both the animals belonging to the surgical group and control group were panting.

#### 2.4.3 Saliva sampling with quantification of salivary vasopressin and cortisol

##### 2.4.3.1 Comparison between brachycephalic dogs with BOAS before and after surgery

###### 2.4.3.1.1 Cortisol

**Figure 5:**
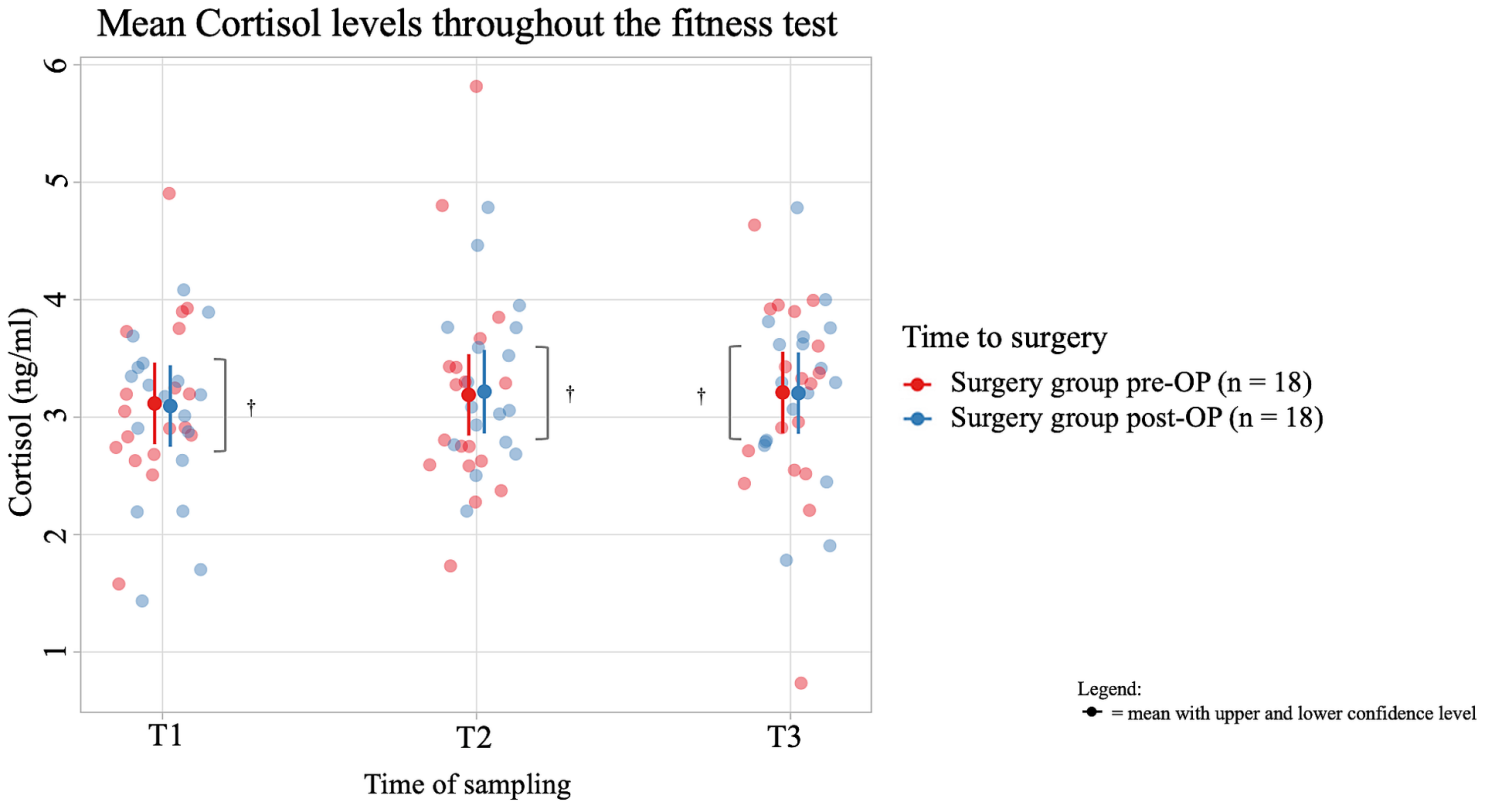
Comparison of the cortisol concentration (in ng/ml) in the saliva within the surgery group animals before and after the BOAS surgery before (T1), immediately after (T2) and 15 min after the fitness test (T3). In the pre-OP group, salivary cortisol increased slightly during and after the fitness test, while in the post-OP group, cortisol initially rose and then slightly decreased, with none of these changes being statistically significant (pre-OP: T1 to T2: *p* = 0.9402, T2 to T3: *p* = 0.9949), post-OP: (T1 to T2: *p* = 0.8459, T2 to T3: *p* = 0.9982). Comparisons between pre- and post-OP groups also showed no significant differences in cortisol levels at any time point (T1: *p* = 0.9204, T2: *p* = 0.9025, T3: *p* = 0.9770). ^†^*p* ≥ 0.05.

The average salivary cortisol concentration of dogs in the pre-OP group increased by 0.0731 ng/mL from T1 (immediately before the fitness test) to T2 (immediately after the fitness test) and by 0.0210 ng/mL from T2 to T3 (15 min after the end of the fitness test), but these changes were not significant (T1 to T2: *p* = 0.9402, T2 to T3: *p* = 0.9949; Figure 5; Supplementary Figure S2). In the post-OP group, the average salivary cortisol concentration initially increased by 0.1226 ng/mL from T1 to T2 of the fitness test and then decreased by 0.0128 ng/mL from T2 to T3. However, these changes were not significant (T1 to T2: *p* = 0.8459, T2 to T3: *p* = 0.9982). Comparing the pre- and post-OP values, the post-OP dogs had a 0.02200 ng/mL lower salivary cortisol concentration before the fitness test than before surgery. Immediately after the fitness test, the average salivary cortisol concentration before surgery was 0.02744 ng/mL lower, only to be 0.00634 ng/mL higher than in the post-OP group 15 min after the fitness test. All values were not statistically significant (T1: *p* = 0.9204, T2: *p* = 0.9025, T3: *p* = 0.9770).

In one dog, saliva sampling was not possible due to severe dyspnea pre-surgery and in 9 samples there was an error in the laboratory analysis, which is why these samples could not be evaluated.

###### 2.4.3.1.2 Vasopressin

**Figure 6:**
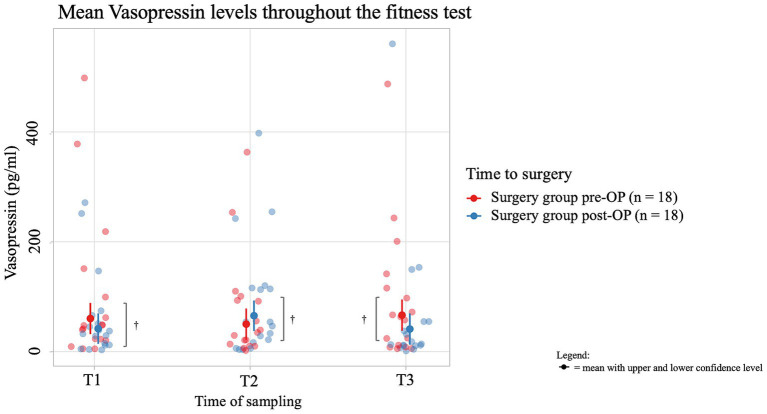
Comparison of vasopressin concentration (in pg/ml) in the saliva within the surgery group animals before and after BOAS surgery before (T1), immediately after (T2) and 15 min after the fitness test (T3). In the pre-OP group, salivary vasopressin decreased during the fitness test and then increased afterward, with changes not reaching statistical significance (T1 to T2: *p* = 0.8379, T2 to T3: *p* = 0.6337). In the post-OP group, vasopressin initially increased during the fitness test and then decreased, also without significant results (T1 to T2: *p* = 0.3667, T2 to T3: *p* = 0.3584). Comparisons between pre- and post-OP groups showed no significant differences at any time point, with the post-OP group having slightly lower vasopressin levels before and after the fitness test and slightly higher immediately after (T1: *p* = 0.2993, T2: *p* = 0.3905, T3: *p* = 0.1615). ^†^*p* ≥ 0.05.

The average salivary vasopressin concentration of dogs in the pre-OP group decreased by 10.184 pg./mL from T1 to T2 of the fitness test and then increased by 16.361 pg./mL from T2 to T3, but these changes were not significant (T1 to T2: *p* = 0.8379, T2 to T3: *p* = 0.6337; Figure 6; Supplementary Figure S3). In the post-OP group, the average salivary vasopressin concentration initially increased by 23.520 pg./mL from time T1 to T2 of the fitness test, only to decrease again by 24.179 pg./mL from T2 to T3. Again, these changes were not significant (T1 to T2: *p* = 0.3667, T2 to T3: *p* = 0.3584). When comparing the values between the groups, it is noticeable that the post-OP animals had a salivary vasopressin concentration 18.4 pg./mL lower than the pre-OP animals before the fitness test. Immediately after the fitness test, the average salivary vasopressin concentration in the post-OP animals increased by 15.3 pg./mL, only to be 25.3 pg./mL lower than in the pre-OP group 15 min after the fitness test. There were no statistically significant differences between pre- and post-OP at any time point (T1: *p* = 0.2993, T2: *p* = 0.3905, T3: *p* = 0.1615). Due to technical problems, one saliva sample was missing in four animals and therefore the vasopressin level was not determined at this time point. In one animal, the pre-OP saliva sample had to be omitted due to dyspnea and any pre-OP vasopressin values were missing.

##### 2.4.3.2 Comparison between brachycephalic dogs with BOAS pre-OP and brachycephalic dogs without BOAS

###### 2.4.3.2.1 Cortisol

**Figure 7:**
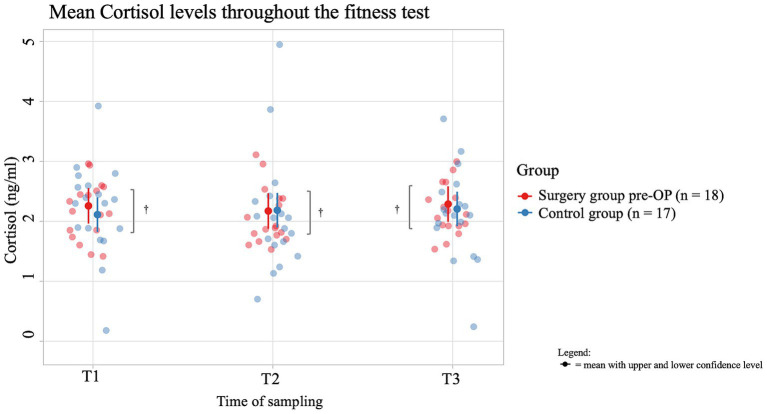
Comparison of the cortisol concentration (in ng/ml) in the saliva within the control group animals and the surgery group animals before the BOAS surgery before (T1), immediately after (T2) and 15 min after the fitness test (T3). In the pre-OP group, salivary cortisol increased slightly during and after the fitness test, but these changes were not statistically significant (T1 to T2: *p* = 0.9402, T2 to T3: *p* = 0.9949). In the control group, cortisol initially decreased during the fitness test and then slightly increased, also without statistical significance (T1 to T2: *p* = 0.6834, T2 to T3: *p* = 0.9568). Comparisons between the control and pre-OP groups at all time points showed differences in cortisol concentrations, but none were statistically significant (T1: *p* = 0.485, T2: *p* = 0.9531, T3: *p* = 0.6971). ^†^p ≥ 0.05.

The average salivary cortisol concentration of dogs in the pre-OP group increased by 0.0731 ng/mL from T1 to T2 of the fitness test and by 0.0210 ng/mL from T2 to T3, but these changes were not significant (T1 to T2: *p* = 0.9402, T2 to T3: *p* = 0.9949; Figure 7). In the control group, the average salivary cortisol concentration initially decreased by 0.0858 ng/mL from T1 to T2 of the fitness test and increased again by 0.0292 ng/mL from T2 to T3. However, these changes were not significant (T1 to T2: *p* = 0.6834, T2 to T3: *p* = 0.9568). At all time points (T1, T2, T3) there was a difference in average cortisol concentrations between the control group and the pre-OP group, which was not statistically significant (T1: *p* = 0.485, T2: *p* = 0.9531, T3: *p* = 0.6971).

###### 2.4.3.2.2 Vasopressin

**Figure 8:**
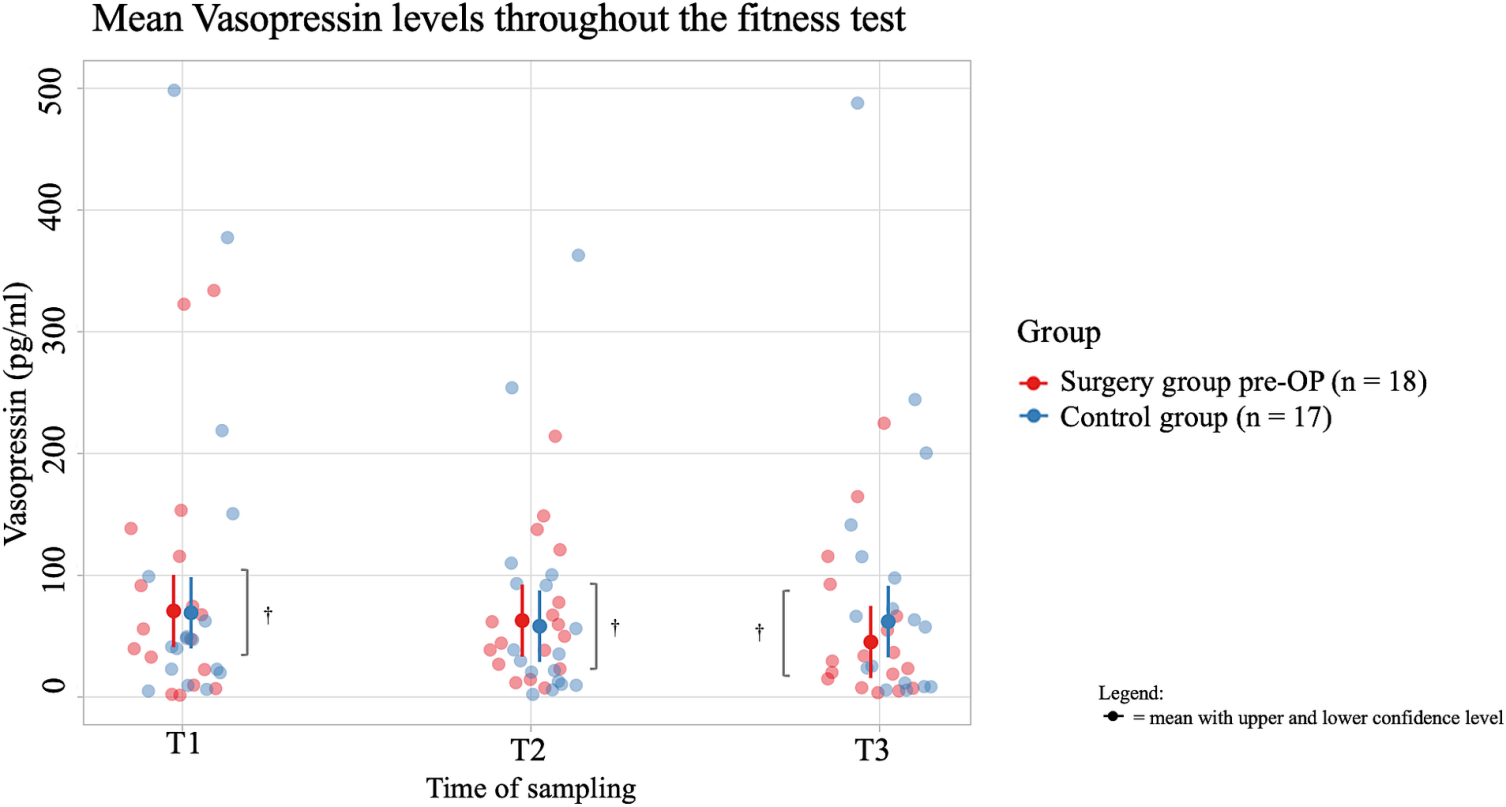
Comparison of the vasopressin concentration (in pg/ml) in the saliva within the surgery group animals pre-OP and the control group animals before (T1), immediately after (T2) and 15 min after the fitness test (T3). In the pre-OP group, salivary vasopressin decreased during the fitness test and increased slightly during the recovery period, with neither change being statistically significant (T1 to T2: *p* = 0.8379, T2 to T3: *p* = 0.6337). In the control group, vasopressin decreased steadily throughout the fitness test and recovery, but these changes were also not significant (T1 to T2: *p* = 0.7112, T2 to T3: *p* = 0.8835). Comparisons between the pre-OP and control groups showed consistently lower vasopressin levels in the control animals, though none of the differences were statistically significant (T1: *p* = 0.6437, T2: *p* = 0.6805, T3: *p* = 0.2466). ^†^*p* ≥ 0.05.

The average salivary vasopressin concentration of dogs in the pre-OP group decreased by 11 pg./mL from T1 to T2 of the fitness test and increased again by 3.8 pg./mL from T2 to T3 during the recovery period, but these changes are not significant (T1 to T2: *p* = 0.8379, T2 to T3: *p* = 0.6337; Figure 8). In the control group, the average salivary vasopressin concentration first decreased by 7.9 pg./mL from T1 to T2 of the fitness test and then by a further 17.6 pg./mL from T2 to T3. However, these changes were not significant (T1 to T2: *p* = 0.7112, T2 to T3: *p* = 0.8835). Comparing the average values between groups, the control animals consistently had lower salivary vasopressin concentrations than the pre-OP group. However, none of these differences were statistically significant (T1: *p* = 0.6437, T2: *p* = 0.6805, T3: *p* = 0.2466).

#### 2.4.4 General fitness on the treadmill

Seventeen control animals completed the entire fitness test, whereas none of the patients in the pre-OP group did (Table 2). Post-OP, two of the 18 patients completed the entire test.

**Table 2:** Difference in the number of dogs that passed the fitness test pre-OP and post-OP.

Time of fitness test (in minutes)	Number of animals performing the fitness test at this time point pre-OP	Number of animals performing the fitness test at this time point post-OP	Number of animals with improvement in the fitness test at this time point*	Change in BOAS-grade
0	18	18	N/A	N/A
2.5	18	18	0	
5	3	6	5	2× 3 → 1 1× 2 → 1 2× 2 = 2
6	2	4	2	2× 2 = 2
8.5	2	4	2	
11	1	4	3	2× 2 = 2 1× 1 = 1
12	0	3	3	2× 3 → 1 1× 2 = 2
14.5	0	2	2	2× 3 → 1
17	0	2	2	

^1^FB = French Bulldog, ^2^EB = English Bulldog. * This is the number of animals that have reached this specific time point post-OP, whereas they have never reached this time point pre-OP.

Pre-OP, 14 patients in the surgery group had to stop the fitness test after 2.5 min, while one patient reached 5 min, 8.5 min and 12 min, respectively. Post-OP, only 11 tests had to be stopped after 2.5 min, while three patients had to stop after 5 min. One test was stopped at 11 min and one at 12 min. The patients who completed the test post-OP had to stop the test pre-OP after 2.5 and 11 min, respectively. In 16 of the patients in the surgery group, tachycardia was present pre-OP. In nine patients, this was accompanied by high-pitched respiratory sounds, and in one patient there was also insufficient willingness to walk. Post-OP, 14 fitness tests were stopped because of tachycardia, and nine of these patients also had high-pitched respiratory sounds. The number of patients who took more than 15 min to fully recover pre-OP was reduced from 15 to nine post-OP. Similarly, five patients recovered after only 5 min post-OP, compared to only one patient pre-OP. Two patients recovered after a maximum of 10 min post-OP and two patients required a maximum of 15 min both pre- and post-OP. The recovery time after completing the fitness test was a maximum of 5 min for six control animals and four control animals recovered after a maximum of 10 min. Seven control animals needed a maximum of 15 min.

## 3 Discussion

Symptoms of BOAS can vary, but often include excessive snoring, noisy or labored breathing and shortness of breath (38). Additional symptoms such as exercise intolerance, choking, vomiting, and collapse are common and, in severe cases, can be life-threatening (6, 38, 39).

In this study, vital signs were measured during the fitness test and a subjective assessment of general health was made as part of a medical history interview. BOAS patients in the surgery group had higher respiratory and heart rates than the control group, consistent with findings by Mach et al. (30), which also investigated fitness tests in brachycephalic dogs. The authors support the use of vital signs during the test to discriminate between dogs with BOAS and those without or with mild disease. Post-OP, the surgery group exhibited lower heart rates, aligning with findings by Žgank et al., (26) where brachycephalic dogs were undergoing similar fitness tests. However, respiratory rates showed no clear change, as panting dogs were assigned a standardized rate of 200 breaths per minute.

Stress parameters, including cortisol and vasopressin, were also measured using saliva samples. Cortisol levels, often indicative of stress, showed no statistically significant differences between study and control groups or between pre- and post-OP measurements (40–42). This could be due to limited valid cortisol data from only eight dogs out of 18, as some samples were unusable due to collection difficulties in one case and analysis issues in nine cases. Furthermore, cortisol levels can be influenced by various factors, such as time of day and individual characteristics, limiting its use as a stand-alone stress indicator (40, 43). Our study builds on previous research that has documented altered cortisol responses in BOAS-affected dogs. Consistent cortisol levels in both groups may suggest HPA-axis dysfunction, potentially linked to chronic stress in brachycephalic dogs, as suggested by Kähler et al. (21). Interestingly, even the control group in our study, consisting of brachycephalic dogs without BOAS surgery, displayed similar patterns of constant cortisol levels. This suggests that the dysregulation of the HPA-axis might not be exclusive to BOAS-affected dogs but could be a broader issue within the brachycephalic population. The consistency of cortisol and vasopressin levels across both groups reinforces the hypothesis of a stress-induced HPA-axis dysfunction, which may contribute to the overall health challenges faced by these dogs, regardless of BOAS-grade (21, 44). Similarly, vasopressin levels showed no significant differences, possibly due to insufficient evaluable samples. Previous research suggests that salivary vasopressin levels are negatively correlated with acute stress situations and that vasopressin levels fluctuate during acute stress, but our findings indicate that these stress hormone levels require interpretation alongside clinical signs and vital parameters (17). The results highlight that parameters such as salivary stress hormones should always be interpreted in combination with a clinical examination, such as stress scoring or vital signs.

Based on the BOAS-grade, eight dogs in this study showed improvement post-OP, improving by at least one grade. According to the clinical interview, 14 dogs had an improvement in clinical symptoms and six dogs were able to perform the fitness test longer than before surgery. Similar results were found in a study by Žgank et al. in which both respiratory problems and fitness improved significantly in French Bulldogs that underwent BOAS surgery (26). Based on the BOAS-grade, 10 dogs showed no improvement or deterioration post-OP. Based on clinical history, two dogs had no improvement or worsening of clinical symptoms, and 12 dogs maintained the fitness test for the same length of time as before surgery. Torrez and Hunt (45) reported no improvement in 11% of operated dogs, while Riecks et al. (46) found no improvement or worsening in 6% and noted that nearly half showed improvement but still had clinical signs. In this study, the symptoms of two patients worsened, and the fitness test had to be stopped after 2.5 min both pre- and post-OP due to dyspnea. In one of these dogs, the respiratory sounds worsened at rest. Rhinoplasty, staphylectomy, tonsillectomy and resection of inverted sacculi were performed. First-degree aberrant conchae, which are conchae that are “visible in the ventral and nasal meatus, but not extending into the nasopharyngeal meatus,” were identified on CT, which could describe a potential cause of the respiratory problems, as the presence of aberrant conchae can increase the air resistance while breathing and lead to reduced ventilation of the sinuses (26, 47, 48). However, this finding was already present pre-OP. A study by Oechtering et al. (49) found that, according to CT scans, 2/3 of brachycephalic dogs had abnormal conchae and that more than 90% of these dogs had mucosal contact of the conches. Furthermore, it was demonstrated that the presence of aberrant conchae could not provide satisfactory results with conventional surgical approaches. This represents a limitation of the study and could explain why the majority of patients continued to suffer from limited fitness despite improvements in their health. In the other dog found to deteriorate post-OP, this was associated with increased exercise intolerance. However, the owner reported an improvement in respiratory sounds and finds that the dogs wellbeing overall has improved post-OP. The pre- and post-OP fitness test were both stopped at the same time due to tachycardia. Rhinoplasty, staphylectomy and resection of redundant nasal folds were performed. A CT scan was not performed at the owner’s request and therefore no information was available on the condition of the conchae. Without a pre-OP CT scan, it’s challenging to assess whether additional upper airway abnormalities contributed to the lack of improvement or worsening of symptoms. Radiographs confirmed the presence of tracheal hypoplasia, a pre-existing condition that may contribute to the observed limitations in fitness by compounding the effects of other respiratory anomalies. As noted by Ginn et al. (47), tracheal hypoplasia can exacerbate clinical signs in brachycephalic dogs and reduce their overall tolerance for physical exertion. However, this finding was already present pre-OP. Reasons for the deterioration in both dogs could be post-OP wound healing issues such as swelling or granulation tissue, which increases respiratory effort during physical activity (5). While granulation tissue and edema are known to develop at different stages post-OP, the timing of this dog’s deterioration, reported at the four-week follow-up, complicates drawing definitive conclusions. To gain more clarity, the dog should be assessed under anesthesia to check for these conditions and evaluate the potential causes of the deterioration. Behavioral factors, such as cautious post-OP activity restrictions by owners, or mood-dependent variations in performance, could also play a role. However, there are no studies on these topics yet, and this could not be proven in detail in this study. The owner’s subjective assessment might also influence the perception of post-OP outcomes. However, this could also not be proven in detail in this study.

Other respiratory conditions may also influence the occurrence of respiratory sounds and exercise tolerance. For example, Lindsey et al. (50) found aspiration pneumonia in 4% of dogs after BOAS surgery and Carabalona et al. (51) found 0.5%. However, this could not be determined in this dog. With a BCS of 7/9, this dog’s prognosis was found to be worse than that of a dog with a normal BCS which is consistent with studies by Liu et al. (28, 52), which identified obesity as a significant BOAS risk factor. The mean BCS in this study’s surgery group (6.4/9) was comparable to the control group (5.6/9). However, we found that the BCS of the dog that worsened in the fitness test (BCS 7/9) was slightly higher than the median BCS of the dogs that improved or had the same results pre- and post-OP (BCS = 6, SD = 1.1127 and BCS = 6, SD = 1.4298 respectively). Understanding how BCS and other factors influence prognosis highlights the need for comprehensive assessment methods for BOAS-affected dogs. In a study by Liu et al., (27) the severity of BOAS was measured in Pugs, French Bulldogs and English Bulldogs using whole-body barometric plethysmography (27). Although the average severity in all three breeds was improved by BOAS surgery, the dogs were still clinically affected post-OP. Young age, normal BCS and the occurrence of laryngeal collapse were considered negative prognostic factors (27, 28, 51). In this study, earlier surgery may have led to a greater improvement in symptoms and physical fitness at follow-up. This has been shown in previous studies (24, 37, 53).

It is worth noting that one dog in this study presented with laryngeal collapse grade 2 and underwent arytenoid lateralization. This surgical intervention was performed in addition to the BOAS-related procedures (rhinoplasty, staphylectomy and resection of everted sacculi). Even though the owner reported an improvement in respiratory signs, the post-OP fitness test could not be performed any longer than pre-OP. A study by Packers and Tivers (5) confirms that factors such as those mentioned above can influence the post-OP outcome and that respiratory symptoms can be alleviated but not eliminated. Even though surgery can immediately improve clinical signs, long-term results are variable with one study showing recurrence of BOAS-associated symptoms and another study showing lasting improvement (45, 54). Torrez and Hunt (45) also found little improvement in clinical signs in almost 1/3 of dogs.

This study has several limitations that may have influenced the findings and their interpretation. A potential factor contributing to the lack of significant improvement in BOAS-grading post-OP may be the extent to which the soft palate is shortened during surgical intervention. Inadequate resection of the soft palate can leave portions of the airway obstructed, thereby hindering expected improvements in clinical symptoms and BOAS-grading. This could explain why a substantial proportion of dogs in this study did not show improvement in their BOAS-grade following surgery. Recent studies have suggested that a more functional soft palate resection technique, such as the folded flap palatoplasty, could better alleviate respiratory clinical signs. For instance, Findji and Dupré (54) reported that almost 98% of dogs showed improvement in respiratory clinical signs within 15 days post-OP. Similarly, Haimel and Dupré (55) documented overall clinical improvement in almost 89% of cases. These findings contrast with the present study, where approximately 55% of dogs remained in the same BOAS-grade post-OP.

It is important to highlight that the small sample size of this study represents a significant limitation. Many owners of brachycephalic dogs expressed concerns about general anesthesia and surgical risks during the history interview and opted against surgery due to the increased risk of complications (56). Furthermore, previous studies have shown that the majority of owners perceive the clinical signs of BOAS as typical breed characteristics rather than problematic or contrary to animal welfare (57). Larger studies with more patients are needed to provide a more robust assessment of the outcomes of BOAS surgery.

Additionally, we did not assess potential correlations between age, weight, and cephalic subtype, which could influence the severity of BOAS-related symptoms and post-OP recovery. It is well established that certain breeds or individual characteristics (such as weight or age) may exacerbate BOAS symptoms or impact the success of surgical outcomes (5). Furthermore, this study did not include an evaluation of nasal turbinates or the presence of tracheal hypoplasia in all the animals. This was due to the fact that a CT scan was not performed in every animal, as some owners chose not to pursue this diagnostic procedure for their pets.

Moreover, since the focus in this study was put on biomarkers of acute stress, such as cortisol and vasopressin, we did not measure biomarkers for chronic and oxidative stress like cortisol in hair, malondialdehyde, prolactin, C-reactive protein, or cardiac biomarkers (58–62). These could provide a more comprehensive understanding of the chronic physiological strain placed on these dogs due to BOAS and its surgical treatment. Blood pressure measurements and temperature evaluations were also not recorded, which would have provided valuable insight into the cardiovascular health of these animals both pre- and post-OP (26, 62).

It can also be assumed that some dogs were stressed in the unfamiliar environment, despite the familiarization period on the treadmill, which appears to have an impact on the release of stress hormones. Specifically, while the control animals did not begin panting until approximately 11 min into the treadmill exercise, the BOAS-affected dogs exhibited panting from the outset of the exercise session, both pre- and post-OP. This early onset of panting suggests that restricted airflow through the nasal passages, characteristic of BOAS, may have contributed significantly to the elevated respiratory rate. Additionally, the potential influence of stress associated with the veterinary environment should not be overlooked, as it could also have played a role in the heightened respiratory rate observed at time point 0. It should also be noted that although the physical fitness of the operated animals improved compared to the pre-OP examination, the patients still showed severe limitations in physical fitness. This suggests that the physical exertion during the follow-up examination was also associated with enormous stress.

It should be noted that the follow-up was deliberately performed just 4 weeks after surgery as most dogs show significant improvement in clinical symptoms, like respiratory distress and exercise intolerance shortly after the surgery. To the author’s knowledge, most literature on this matter describes the long-term effect of the BOAS surgery on affected brachycephalic dogs (45, 46, 63). Because of the grave impact of this condition on the health, quality of life and life span of affected dogs, the goal of treatment should be an immediate alleviation of associated symptoms which can already be seen in our study (23, 24). Additionally, it can be assumed that an improvement in training condition can occur with an increase in exercise. The results of Žgank et al. (26) support this assumption with dogs having a lower heart rate, lower body temperature, fewer respiratory problems and a shorter recovery time after the fitness test at check-up that were carried out six to 9 months post-OP. On average, all in all exercise tolerance was significantly improved. It is very important that the fitness test is carried out by trained personnel who are experienced with handling brachycephalic dogs and who are critical of the animals’ fitness.

In summary, while BOAS surgery can provide slight relief from some symptoms, patients still experience significant limitations in physical fitness. No statistically significant changes were observed in cortisol and vasopressin concentrations in saliva, indicating that the hormonal stress response did not show substantial alterations following the surgery. This could be related to the fact that while the surgery improves certain aspects of the disease, it does not entirely resolve the complex underlying pathology of BOAS, which might explain why physical fitness remains compromised in some animals despite surgical intervention. The relationship between surgery, fitness, and vital signs requires further exploration, and future studies with larger sample sizes, more comprehensive imaging, and additional biomarkers may provide deeper insights into the long-term benefits and challenges of treating BOAS in brachycephalic dogs.

## Data Availability

The original contributions presented in the study are included in the article/Supplementary material, further inquiries can be directed to the corresponding author/s.
