# Supplementary material for: Short-term effects of brachycephalic obstructive airway syndrome surgery on fitness and exercise in brachycephalic dogs
Source: Front Vet Sci. 2025 Feb 18;12:1481717. doi: 10.3389/fvets.2025.1481717 (PMC11876419; doi:10.3389/fvets.2025.1481717)
Supplement: Supplementary file 1 [file Table_1.DOCX]

Supplementary Material

## Supplementary Figures


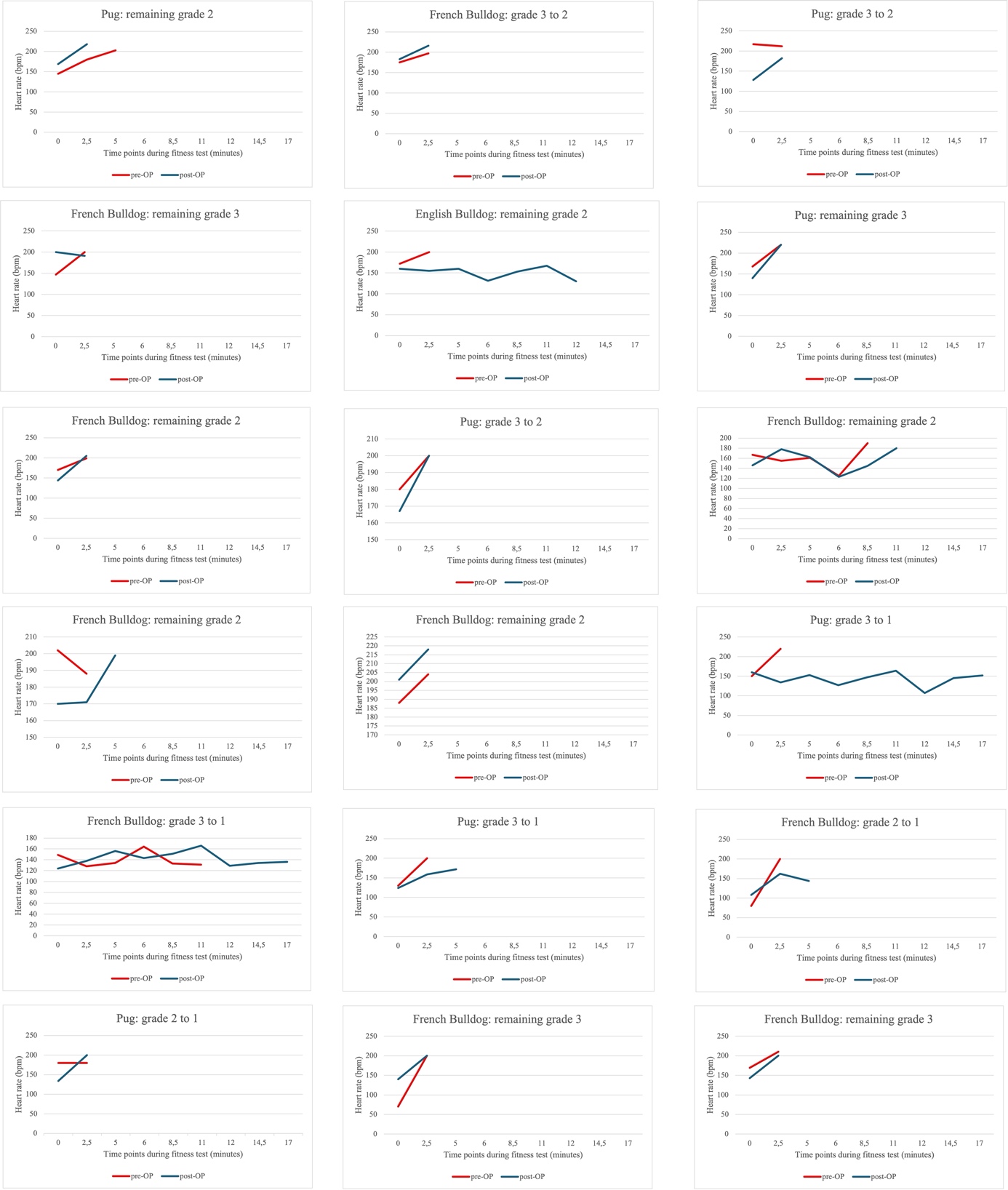


**Supplementary Figure 1.** Progression of the individual heart rate pre-OP compared to post-OP per time point in the fitness test


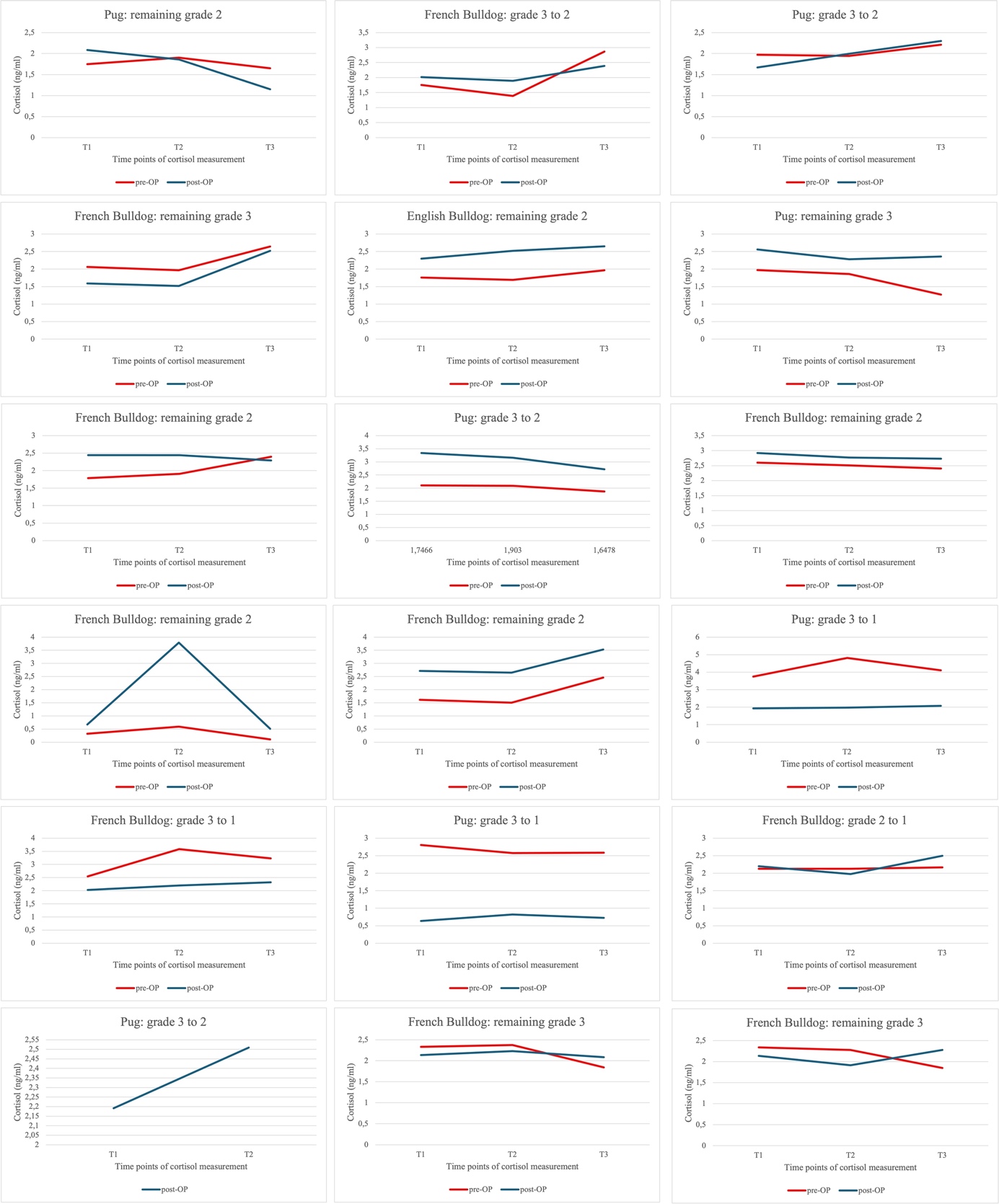


Supplementary Figure 2. Progression of individual cortisol concentrations pre-OP compared to post-OP per time point in the fitness test


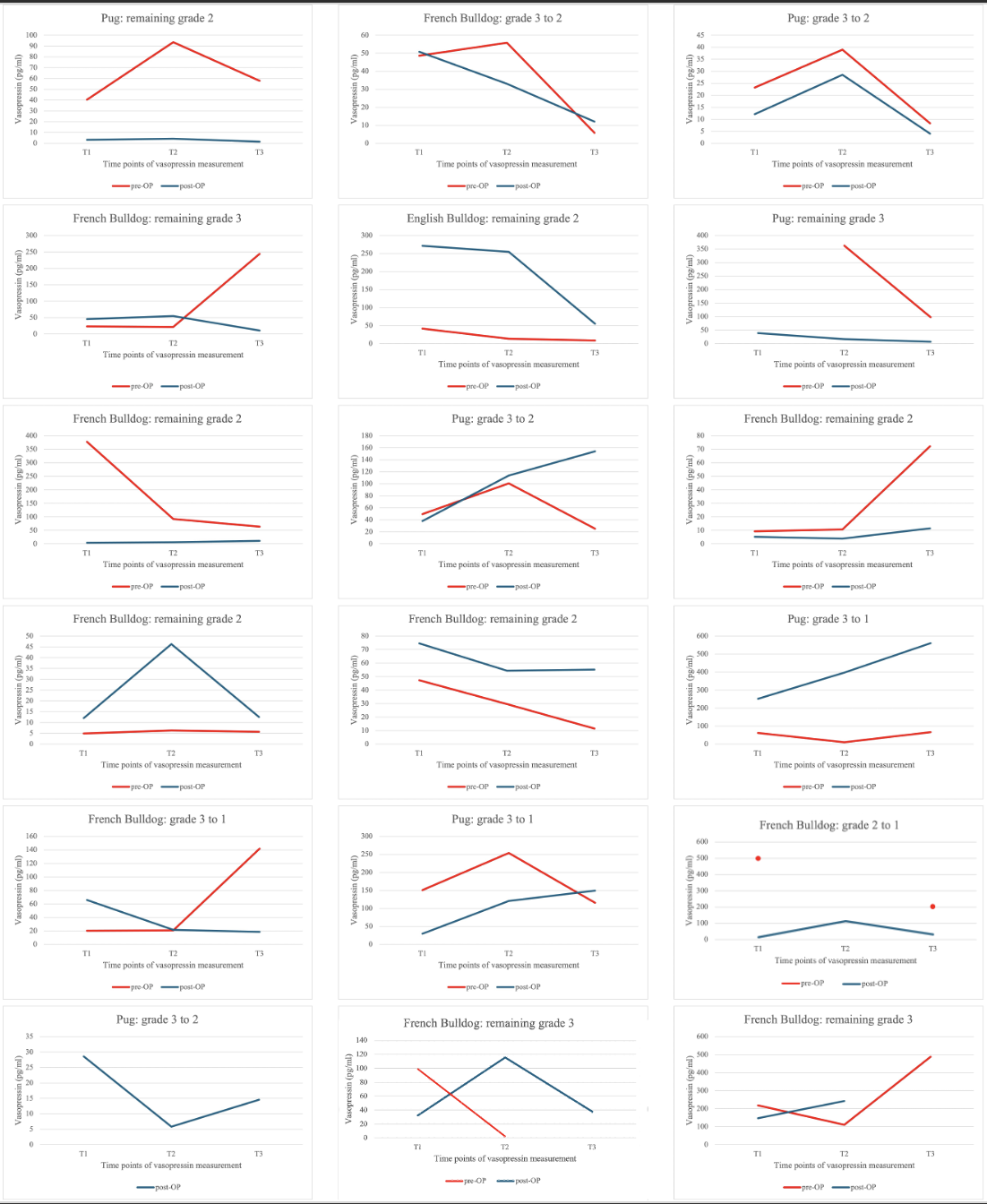


Supplementary Figure 3. Progression of individual vasopressin concentrations pre-OP compared to post-OP per time point in the fitness test
